# Molecular Genetics of β-Cell Compensation in Gestational Diabetes Mellitus: Insights from CDKAL1, SLC30A8 and HHEX

**DOI:** 10.3390/ijms27021121

**Published:** 2026-01-22

**Authors:** Justyna Hryniewicka, Angelika Buczyńska-Backiel, Monika Zbucka-Krętowska, Adam Jacek Krętowski, Małgorzata Szelachowska

**Affiliations:** 1 Department of Endocrinology, Diabetology and Internal Medicine, Medical University of Bialystok, ul. M. Sklodowskiej-Curie 24a, 15-276 Bialystok, Poland; adamkretowski@wp.pl (A.J.K.); malgorzata.szelachowska@umb.edu.pl (M.S.); 2 Clinical Research Centre, Medical University of Bialystok, ul. M. Skłodowskiej-Curie 24a, 15-276 Bialystok, Poland; 3 Department of Gynecological Endocrinology and Adolescent Gynecology, Medical University of Bialystok, ul. M. Skłodowskiej-Curie 24a, 15-276 Bialystok, Poland; monikazbucka@wp.pl

**Keywords:** gestational diabetes mellitus, maternal-fetal imbalances, genetic patterns, genetic determinants, molecular mechanisms

## Abstract

Gestational diabetes mellitus (GDM) is a common metabolic complication of pregnancy associated with significant short- and long-term risks for both mother and offspring. Increasing evidence indicates that genetic susceptibility plays a central role in GDM pathogenesis, particularly through variants affecting insulin secretion and pancreatic β-cell function. This narrative review integrates molecular, clinical, and epidemiological perspectives, highlighting population-specific effects and gene–environment interactions. Improved understanding of the genetic risk architecture may support earlier risk stratification and enable the future development of personalized strategies for GDM prevention and management, with particular emphasis on genetic polymorphisms in *SLC30A8*, *CDKAL1*, and *HHEX* genes consistently implicated in glucose homeostasis and β-cell integrity. These genes contribute to distinct but complementary molecular pathways underlying GDM, including impaired insulin biosynthesis, defective zinc transport within insulin granules, and altered paracrine regulation within pancreatic islets. While associations between these variants and GDM have been repeatedly demonstrated, their clinical relevance and mechanistic impact remain incompletely understood. Available evidence suggests that *CDKAL1* represents the strongest genetic determinant, followed by *SLC30A8*, while *HHEX* appears to play a modulatory role. This review summarizes current findings on the molecular functions and clinical significance of these polymorphisms, highlighting population-specific effects and gene–environment interactions. Improved understanding of genetic risk architecture may support earlier risk stratification and enable future development of personalized strategies for GDM prevention and management.

## 1. Introduction

Gestational diabetes mellitus (GDM) is among the most frequently diagnosed metabolic disorders during pregnancy, accounting for approximately 88% of all diabetes cases in pregnant women. The remaining 12% are attributed to type 2 diabetes (T2D) and type 1 diabetes (T1D) [1]. GDM is defined as varying degrees of glucose intolerance identified or first detected during pregnancy, with most cases resolving postpartum [2,3]. This definition of GDM does not exclude the possibility of previously undiagnosed diabetes but does exclude diabetes diagnosed prior to pregnancy [2]. The prevalence of GDM generally mirrors the prevalence of T2D and is estimated to range between 4% and 14%, depending on the population studied and the diagnostic criteria used. In the United States, ethnic groups at the highest risk for GDM include Hispanic, Asian, Native American, and African American women. In Europe, GDM is more prevalent among Asian women compared to Caucasian women. In most countries, the prevalence of GDM is estimated at 2% to 6% of all pregnancies [4,5,6,7]. For the Polish population, the prevalence was approximately 1% to 3% in 2002 [8].

GDM is associated with significant consequences for both the mother and the developing fetus. Maternal hyperglycemia leads to fetal hyperglycemia because glucose readily crosses the placenta. In response to elevated glucose levels, the fetal pancreas produces and secretes increased amounts of insulin. Fetal hyperinsulinemia is responsible for many perinatal complications associated with GDM. One of the major fetal complications associated with GDM is excessive fetal growth, commonly reported as fetal macrosomia or large-for-gestational-age depending on the definition used (absolute thresholds or gestational-age–adjusted percentiles) [9,10]. The disproportionate, rapid growth results in increased subcutaneous fat accumulation and an elevated risk of perinatal injuries. Newborns of mothers with diabetes are more predisposed to developing respiratory distress syndrome and hypoglycemia in the early postpartum period, as well as hyperbilirubinemia, hypocalcemia, and hypomagnesemia [11,12,13]. Research performed by Wahabi et al. has demonstrated that GDM and maternal obesity are positively correlated with increased birth weight of the offspring and the necessity for cesarean delivery [14]. Children born to mothers with GDM are at higher risk for developing T2D and obesity. Studies have shown that these children exhibit higher body mass index (BMI) at ages 4 and 7 compared to children of non-diabetic mothers [15].

Numerous studies highlight the significant role of genetic predispositions in the development of GDM. However, only a limited number of genetic polymorphisms have been investigated to date, and there is insufficient understanding of how these polymorphisms affect clinical characteristics.

Among the genetic polymorphisms associated with GDM, variants in the Solute Carrier Family 30 Member 8 (SLC30A8), CDK5 Regulatory Subunit-Associated Protein 1-Like 1 (CDKAL1), and Hematopoietically Expressed Homeobox (HHEX) genes have drawn particular attention. These genes have been implicated in insulin secretion, pancreatic beta-cell function, and glucose homeostasis, key processes that are disrupted in GDM. However, their exact influence on the clinical manifestations of GDM remains insufficiently explored. This review aims to synthesize current evidence on the role of genetic polymorphisms in *SLC30A8*, *CDKAL1*, and *HHEX* in the development of gestational diabetes mellitus, with a particular focus on how these variants disrupt successive stages of β-cell function, from insulin biosynthesis and granule maturation to intra-islet regulation of secretion. By integrating molecular, clinical, and epidemiological data, this review seeks to define a coherent genetic framework for β-cell failure in pregnancy and to clarify how these variants shape individual susceptibility to GDM.

## 2. Materials and Methods

**Figure 1:**
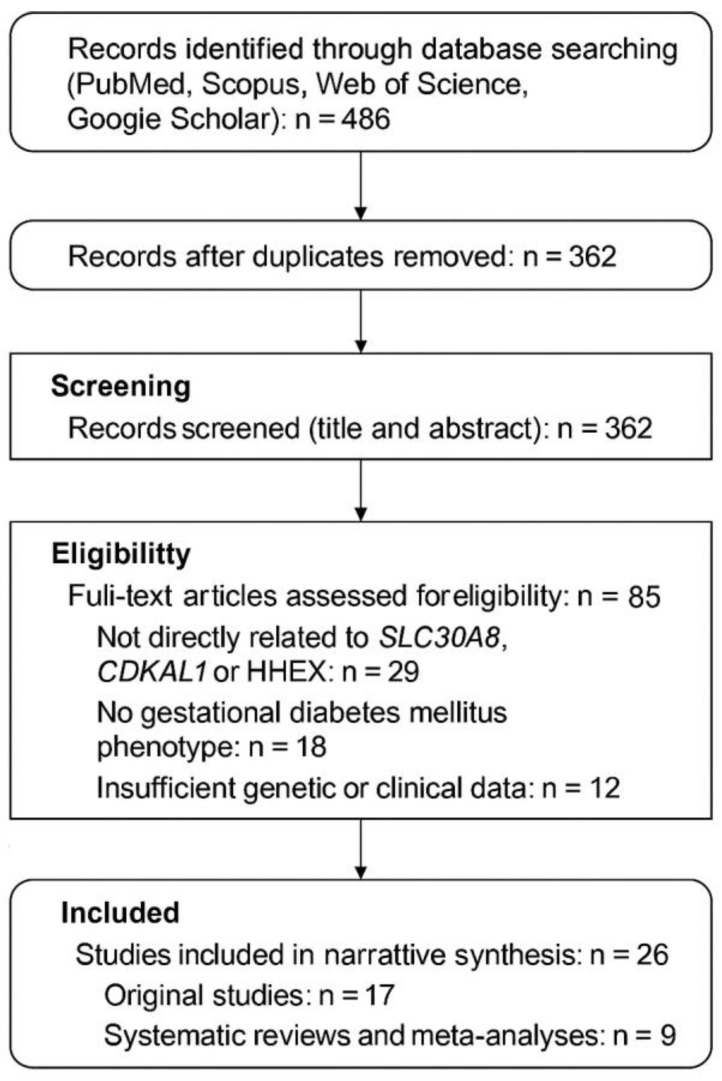
PRISMA flow diagram illustrating the literature identification and selection process. Selected elements of the PRISMA 2020 framework were applied to systematize the literature search within a narrative review.

A comprehensive literature search was conducted to identify studies examining the association between genetic variants in the SLC30A8, CDKAL1, and HHEX genes and the development of GDM. Electronic databases including PubMed, Scopus, Web of Science, and Google Scholar were systematically searched for articles published between January 2000 and December 2024 using combinations of Medical Subject Headings (MeSH) and free-text terms such as “gestational diabetes mellitus,” “GDM,” “genetic polymorphism,” “single nucleotide polymorphism,” “SLC30A8,” “CDKAL1,” “HHEX,” “beta-cell dysfunction,” “insulin secretion,” and “glucose metabolism.” Only studies published in English were included. Original research articles, systematic reviews, and meta-analyses involving human subjects were considered eligible if they specifically evaluated the association between the selected gene variants and GDM. Animal studies, case reports, abstracts without full text, and studies not directly addressing GDM were excluded unless they provided essential mechanistic insights supporting clinical interpretation. Titles and abstracts were screened independently by two authors, followed by a full-text review of potentially relevant articles. Data extracted from each eligible publication included study design, population characteristics, sample size, diagnostic criteria for GDM, investigated gene polymorphisms, and reported clinical or functional outcomes. The results were synthesized using a narrative approach focusing on biological relevance and consistency across studies. Study quality was assessed using the Newcastle–Ottawa Scale for observational studies and PRISMA recommendations for meta-analyses. Only studies of moderate to high methodological quality were considered during interpretation. As this review is based solely on previously published data, ethical approval and informed consent were not required. Limitations of this approach included heterogeneity in diagnostic criteria, population differences, and variability in study design, which may have influenced data interpretation. Selected elements of the PRISMA 2020 guidelines were applied to transparently organize the literature identification, screening, and selection process. The review was conducted as a narrative synthesis, and the PRISMA framework was used to systematize inclusion of core genetic studies, while additional references were cited to provide epidemiological, clinical, and mechanistic background (Figure 1) [16]. Quantitative evidence from key genetic association studies was summarized in a structured evidence table to enable transparent comparison of study populations, investigated variants, effect estimates, and methodological quality (Table S1).

## 3. Diagnosis

The diagnostic criteria for GDM have evolved substantially over time. Early clinical observations described diabetes emerging during pregnancy and resolving postpartum, and associated it with adverse obstetric outcomes such as miscarriage and macrosomia [17,18]. The concept of GDM and its diagnostic framework were developed further in the mid-20th century, culminating in the 3 h 100 g OGTT criteria proposed by O’Sullivan and Mahan in 1964 and later revised by the National Diabetes Data Group and by Carpenter and Coustan [19,20,21,22].

Current international practice largely follows recommendations introduced by the International Association of Diabetes and Pregnancy Study Groups (IADPSG) based on the HAPO study, which support universal screening with a 75 g 2 h OGTT at 24–28 weeks of gestation and diagnostic thresholds of fasting ≥92 mg/dL, 1 h ≥ 180 mg/dL, or 2 h ≥ 153 mg/dL [2,23,24]. In Poland, national recommendations transitioned from the PTD 2011 protocol to the WHO 2013/IADPSG-based criteria adopted in 2014 [25] (Table 1).

## 4. GDM Pathogenesis

A physiological pregnancy is characterized by a series of metabolic changes influenced by various hormones, including prolactin and placental hormones such as human placental lactogen (hPL), progesterone, estrogens, and human placental growth hormone (hPGH). During the first trimester of pregnancy, there is normally an increased sensitivity to insulin and an elevation in insulin secretion. In the second trimester, however, there is a progressive reduction in tissue sensitivity to insulin, development of hyperinsulinemia, and an increase in adipose tissue. By the third trimester, insulin resistance reaches levels comparable to those observed in T2D.

The pathogenesis of insulin resistance may involve placental hormones and increased expression of adipokines in adipose tissue, such as leptin, resistin, and visfatin. In later stages of pregnancy, there is also an intensified lipolysis, increased postprandial levels of free fatty acids, and a reduction in maternal adipose tissue reserves. These changes result in greater availability of substrates necessary for fetal development. The later stages of pregnancy are also associated with a reduced capacity for glucose production via gluconeogenesis, which is somewhat related to decreased production of glucagon, norepinephrine, and cortisol under hypoglycemic conditions [1,26,27].

Increasing insulin resistance is an adaptation of the maternal organism to the needs of the developing fetus. However, when insulin secretion is insufficient and cannot compensate for the rising insulin resistance, GDM develops [1].

Decreased insulin sensitivity, or increased insulin resistance, is defined as a reduction in the effect of insulin on target organs such as skeletal muscle, liver, and adipose tissue. Obesity is one of the primary causes of insulin resistance. Two types of insulin resistance are distinguished. The first type is pre-receptor insulin resistance, associated with the presence of specific autoantibodies, including those directed against glutamic acid decarboxylase (anti-GAD), tyrosine phosphatase (anti-IA2), and insulin itself. The second type is post-receptor insulin resistance, characterized by defects in the insulin signaling pathway.

During pregnancy, the most significant cause of reduced tissue sensitivity to insulin is considered to be impaired post-receptor mechanisms, resulting from compromised mobilization of the glucose transporter GLUT-4. In women with GDM, there is approximately a 65% decrease in glucose transport and significant disturbances in cell function [28,29,30,31].

Blood glucose levels and their metabolism are regulated by the insulin signaling pathway. Insulin, secreted by beta cells, is a polypeptide consisting of 51 amino acids, composed of A and B subunits linked by disulfide bonds. It binds to a specific receptor on the cell surface. The insulin receptor is a heterotetrameric protein consisting of two extracellular α subunits and two transmembrane β subunits connected by disulfide bonds. Binding of insulin to the α subunits leads to conformational changes that activate the β subunits, which possess tyrosine kinase activity and undergo autophosphorylation of three tyrosine residues (Tyr-1158, Tyr-1162, Tyr-1163). This activation results in rapid phosphorylation of docking proteins such as insulin receptor substrates (IRS) and members of the Shc protein family [32,33,34].

There are four known IRS family proteins, with IRS-1 and IRS-2 playing a crucial role in glucose metabolism regulation. Phosphorylated tyrosine residues on IRS-1/2 serve as binding sites for additional signaling proteins containing SH-2 domains, such as phosphoinositide 3-kinase (PI3K). PI3K consists of two subunits: the regulatory p85, which binds to IRS, and the catalytic p110. Upon binding of p85 to IRS-1/2, there is an increase in the catalytic activity of p110 and subsequent phosphorylation of phosphatidylinositol (4,5)-bisphosphate (PIP2) at the 3′ position in the inositol ring, leading to the formation of Phosphatidylinositol (3,4,5)-trisphosphate—a signaling substrate that recruits tyrosine kinases such as PDK-1, PKB/Akt, and PKC to the cell membrane. Activation of these kinases leads to a series of events, including the translocation of GLUT4 to the cell membrane, glucagon synthesis, and lipogenesis. Additionally, insulin can activate mitogen-activated protein kinase (MAPK) via the PI3K pathway, thereby enhancing the expression of various genes associated with cell proliferation and differentiation [32,33,34].

## 5. Background of Genetic Susceptibility to GDM

The primary question to address in investigating the genetic predispositions to GDM is whether GDM represents a distinct pathological entity or a metabolic condition that identifies women at risk for the development of T2D. As previously noted, the definition of GDM does not preclude the existence of previously undetected carbohydrate metabolism disorders [35].

Although genetic variants associated with type 1 and T2D may also be implicated in GDM, delineating specific genetic variants that predispose to GDM is particularly challenging. Nevertheless, there may be unique interactions between the state of pregnancy and specific genetic variants, which could modify the risk of developing GDM in individuals carrying certain genetic polymorphisms affecting various metabolic pathways [35]. A further complication in genetic research on GDM predisposition is the evolving and non-standardized diagnostic criteria.

The relationship between T2D and GDM, including shared pathogenic mechanisms, similar risk factors, and familial clustering, has led to investigations into the association between polymorphisms related to T2D risk and GDM risk. Research has concentrated on genetic polymorphisms (single nucleotide polymorphisms, SNPs) in genes or adjacent to genes involved in insulin biosynthesis and secretion, as well as those associated with insulin resistance and obesity.

One of the most extensively studied genes associated with insulin secretion is TCF7L2. Numerous studies have demonstrated an association between the presence of risk alleles rs7903146-T and rs12255372-T with GDM. Data from the HAPO study indicate that in the European population, carrying the T allele of the rs7903146 variant was associated with an increased risk of GDM, as well as an elevation in fasting glucose by 0.2 mmol/L and a 0.16 mmol/L increase in glucose levels at the first hour of the oral glucose tolerance test (OGTT). In the Greek population, the presence of the T allele of rs7903146 approximately doubled the risk [OR 2.04 (95% CI 1.38–3.00)] [36]. Other gene variants involved in regulating insulin secretion and significantly associated with GDM include genetic polymorphisms in genes or near genes such as KCNJ11, CDKAL1, MTNR1B, HHEX, SLC30A8, CDKN2A/2B, and IGF2BP2 [37].

Another gene frequently investigated in the context of GDM is the glucokinase gene (GCK). This gene is part of the glucose homeostasis regulatory gene group. The association between the rs1799884 variant (also known as 230G/A) of this gene and GDM has been studied across various populations, although results have been inconsistent. Initial studies conducted by K. C. Chiu et al. in 1994, and subsequently by Zaidi et al. in 1997, found no association between rs1799884 and the incidence of GDM [38,39]. However, later studies involving larger populations have demonstrated an association between this GCK genetic polymorphism and GDM. A meta-analysis of these studies indicated that carrying the T allele of rs1799884 results in approximately a 30% increased risk of GDM [37].

Among the genes associated with insulin resistance studied in GDM populations is PPAR gamma. Eight different studies examined the potential association between rs1801282 and GDM, but no evidence was found to suggest that this variant increases the likelihood of developing the condition. Another gene of interest in this category is IRS1. A meta-analysis of four studies revealed that the T allele of rs1801278 was associated with approximately a 1.39-fold increased risk of GDM [37].

A gene associated with obesity that has been studied in the context of GDM is the FTO gene. Several studies have also investigated variants of genes involved in other metabolic pathways. Notable examples include the beta-adrenergic receptor gene (ADRB3) and the tumor necrosis factor (TNF) gene. For the ADRB3 gene, results from five studies were contradictory [37]. A meta-analysis based on these studies found no association between the rs4994 (Trp64Arg) variant and GDM.

Similarly, no association has been demonstrated between variants of TNF-α or TNF-β and GDM [40].

## 6. SLC30A8

The SLC30A8 gene is located on chromosome 8 at position 8q24.11. It consists of 8 exons and has a length of 37 kb. The SLC30A8 gene was first sequenced in 2004 by Chimienti et al., who isolated RNA from human pancreatic islet cells and performed RT-PCR. This team proposed both the name and symbol for the gene. They also provided the likely structure of the gene product, which is a protein composed of 369 amino acids, featuring 6 transmembrane helices, histidine-rich loops between helices 4 and 5, several phosphorylation sites, 2 N-glycosylations, and 2 N-myristoylations [41]. Expression studies in 24 human tissue samples showed maximal expression in the pancreas. Research using insulinoma cell lines demonstrated that ZNT8 expression changes were dependent on insulin secretion from beta-cell granules (OMIM 611145).

ZNT8 is a member of the zinc transporter protein family, involved in intracellular zinc homeostasis and the proper regulation of zinc ion concentrations within beta cells. Zinc ion concentrations are regulated by importers (ZIP/SLA39), exporters (zinc transporters (ZNT)/SLC30), and metal-binding proteins like metallothioneins (MT). The strongest expression of SLC30A8 is observed in alpha and beta cells of pancreatic islets, with smaller amounts found in the testes and submandibular salivary glands. ZnT-8 is localized in insulin secretory granules, where it, along with ZnT-5, is responsible for the proper maturation (hexamerization), storage, and secretion of insulin. The highest zinc concentration is found in beta cells, where it acts paracrinely on the pancreas, is co-secreted with insulin from beta cells, exerts insulin-mimetic and antioxidant effects, and participates in the regulation of beta-cell mass. Zinc is thought to positively regulate K-ATP channels, which are crucial for proper insulin secretion in response to glucose. It also has a suppressive effect on glucagon secretion [42,43,44].

Zinc and disturbances in its homeostasis are associated with the pathogenesis of T2D. Research conducted by Y. Fu et al. demonstrated that reduced expression of the SLC30A8 gene in INS-1 cell lines decreases insulin content in granules and its secretion in response to glucose (glucose-stimulated insulin secretion index, GSIS). Interestingly, studies performed on mice with a complete knockout of the SLC30A8 gene showed that ZnT8 is not essential for glycemic control. These mice exhibited glucose intolerance that was age-, sex-, and diet-dependent. In contrast, in a model of mice with a targeted knockout of ZnT8 specifically in β-cells of the Langerhans islets (Znt8BKO), there was a reduction in the expression of key transcription factors (PDX1, MAFA) and enzymes involved in insulin production. These mice demonstrated impaired first-phase insulin secretion in response to glucose and glucose intolerance [44,45].

B. Lefebvre et al. found in studies using beta-cell cultures that exposure to fatty acids and zinc chelation resulted in a significant decrease in SLC30A8 expression. Metabolic stress associated with T2D and reduced expression of the investigated gene were significantly correlated with increased basal insulin secretion [42].

Genome-wide association studies (GWAS) have shown a very strong association between polymorphisms (SNPs) of the SLC30A8 gene and T2D across various populations. The first studied polymorphism of the SLC30A8 gene is rs13266634. This missense mutation in the exon results in a nucleotide change from C to T (C/T). This mutation leads to an amino acid substitution: tryptophan is replaced by arginine at position 325 in exon 13 (Exon R [Arg] → W [Trp]). The allele frequencies in the European population according to HapMap CEU are as follows: genotype CC occurs with a frequency of 0.566, CT with 0.389, and TT with 0.044. The A allele is present in 0.761 of Europeans, while the C allele is present in 0.239. The risk allele for T2D is the primary (wild type) C allele (R, Arg). The second polymorphism of SLC30A8 is rs11558471, which is also located in exon 13, within the 3′-UTR region. This polymorphism involves the substitution of nucleotide A (ancestral allele) with G. Following transcription, this polymorphism causes a change in the mRNA within the untranslated region 3′-UTR. The genotype frequencies in the European population, according to HapMap-CEU, are AA with 0.540, AG with 0.416, and GG with 0.044.

The allelic frequencies are as follows: allele A is present at a frequency of 0.748, and allele G at 0.252. The risk allele for T2D, as confirmed by research, is allele A (wild type). Variants rs13266634 and rs11558471 are located in the coding regions of the DNA. This led to the hypothesis that nucleotide sequence alterations caused by the presence of genetic polymorphisms may directly affect the ability of ZnT8 to properly transport zinc ions. Studies conducted by J. T. Nicolson et al. and I. Kim et al. demonstrated that the presence of the T allele (Trp) at rs13266634 is associated with increased zinc uptake by ZnT8. The protective effect of the Trp allele at rs13266634 is attributed to higher transporter activity compared to the primary Arg allele [43,44,46,47].

Interestingly, research by N. M. Maruthur showed that carriers of the RW/WW genotype exhibit reduced insulin secretion in response to glucose compared to RR homozygotes. However, following zinc supplementation, a 15% increase in insulin secretion was observed in the RW/WW group compared to the RR group. Additionally, the RW/WW group showed allele-dependent reductions in the proinsulin ratio, reflecting improved insulin action [48]. A meta-analysis led by S. Kanoni et al. indicated that increased total zinc intake is associated with lower fasting glucose levels. There is an interaction between zinc intake and the rs11558471 variant of SLC30A8. In carriers of the risk allele A, which increases glycemia, an inverse correlation between zinc intake and glucose levels was observed [49].

In patients with T1D carrying the TT genotype at rs13266634, a 45% to 60% decrease in C-peptide secretion was observed 6 and 12 months post-diagnosis, respectively, compared to carriers of the CC and CT genotypes in a recessive model [50,51].

## 7. CDKAL1

**Figure 2:**
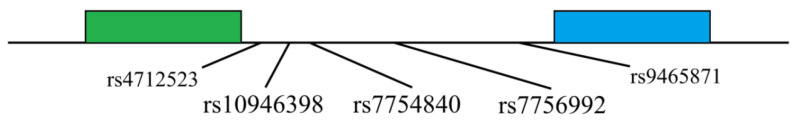
Genetic variants of CDKAL1.

The gene CDKAL1 is located on chromosome 6 at locus p22.3, has a length of 698 kb, and encodes a protein with a mass of 65 kDa. Genome-wide association studies have identified CDKAL1 variants as associated with T2D. In both European and Asian populations, five genetic polymorphisms of this gene, all located in intron 5, have been associated with the disease (Figure 2). Significant associations have been observed between T2D and the rs10946398, rs7754840, and rs7756992 variants in the Han Chinese population [52], as well as rs10946398 and rs7754840 in the African American population [53]. Similar associations have been detected in studies conducted in Japan, Israel, and Korea [54]. The variant examined in this study is rs10946398. Located in intron 5, it involves a nucleotide substitution from C (ancestral allele-C) to A [55]. The frequency of individual genotypes in the European population is as follows: genotype AA 0.425, AC 0.478, and CC 0.097, while the allelic frequencies are: allele A 0.664 (major allele) and allele C 0.336 (minor allele) (HapMap-CEU). The risk allele, as confirmed by GWAS studies, is allele C (risk allele).

Figure 2 illustrates the genetic variants of the CDKAL1 gene, with a focus on the polymorphisms identified in association with T2D. The variants are located in intron 5 of the gene, and the diagram includes their nucleotide positions and substitutions (Figure 2).

CDKAL1 shares a similar amino acid sequence with CDK5 regulatory subunit-associated protein 1 (CDK5RAP1), which binds the regulatory subunits p35 and p39 of CDK5, inhibiting its activity. It has been demonstrated that CDK5 inhibits insulin secretion in response to glucose in beta cells, suppresses insulin gene expression, and leads to beta-cell dysfunction, predisposing to diabetes [56,57].

In 2012, S. Brambillasca et al. [58] discovered that CDKAL1 is a membrane protein localized in the endoplasmic reticulum, belonging to the Tail-Anchored Membrane Proteins (TA) family. Its localization suggests involvement in the translation of secretory proteins. In the INS-1 cell line, decreased expression of CDKAL1 resulted in reduced expression of insulin granule precursor proteins. They also identified an isoform of CDKAL1 and noted that genetic variants might contribute to changes in alternative splicing.

Subsequent studies have shown that CDKAL1 does not interact with CDK5 activators and does not affect its activity. The primary structure of CDKAL1 is characteristic of the MTT-ase family (methylthiotransferase), a subclass of the large AdoMet enzyme superfamily. The MTT-ase family catalyzes the reaction that converts a C-H group to C-SCH3, a process critical for tRNA and ribosomal protein modifications. CDKAL1′s amino acid sequence is similar to MiaB and YqeV, which are part of the MMT-ase family. CDKAL1 catalyzes the addition of a 2-methylthiol group (ms2) to N6-threonyl-carbamoyladenosine (t6A), producing 2-methylthio-N6-threonyl-carbamoyladenosine (ms2t6A) at position 37 of tRNALys(UUU). This methylthiolation is crucial for proper codon recognition of lysine (AAA and AAG) by stabilizing the codon-anticodon binding. This mechanism is essential for proinsulin synthesis, as the absence of CDKAL1 impairs translation and proinsulin production. Disruptions in this methylthiolation process are associated with impaired insulin secretion in T2D [59,60].

Studies have shown that mice with a knockout of the CDKAL1 gene (CDKAL1 KO) exhibit impaired first-phase insulin secretion, while the second phase remains unaffected. This impairment in the first phase is associated with delayed and slow increases in calcium ion concentrations during glucose stimulation, potentially related to defective K+ATP channel responses to glucose [61,62,63].

In studies conducted by Bo Zhou et al., the relationship between CDKAL1 variants and mechanisms potentially leading to the development of T2D was investigated. The focus was on the rs10946398 variant, selected due to its strong linkage with other genetic polymorphisms influencing diabetes susceptibility. The study observed a slight reduction in mRNA expression in carriers of the risk allele. CDKAL1 has two splice variants, and the presence of rs10946398 was associated with a significant decrease in the expression of the first variant, Cdkal1-ν1. Functional studies demonstrated that Cdkal1-ν1 is a non-coding transcript that indirectly regulates CDKAL1 levels by activating CDKAL1-specific miRNAs. SNPs located in intron 5 significantly affected alternative splicing, leading to a reduced level of Cdkal1-ν1, which resulted in weakened miRNA activity. This suppression led to decreased ms2-modification in tRNALys(UUU), causing beta-cell dysfunction. CDKAL1 variants are associated with impaired insulin secretion [60].

## 8. HHEX

The HHEX gene is located on chromosome 10 at position 10q23.33. It consists of 4 exons and spans 5.7 kb. First fully isolated and sequenced in 1992 by M. R. Crompton et al. from a promyelocytic leukemia cell line, HHEX encodes a 270-amino-acid protein with a mass of 30 kDa. It shows high homology across different species [64].

HHEX is a transcription factor containing a DNA-binding domain known as a homeodomain. Like most homeotic genes, HHEX regulates development through mechanisms of transcriptional regulation and post-transcriptional modifications, and influences cell differentiation. Some of the genes regulated by HHEX encode proteins involved in cell cycle control or growth factor signaling pathways. It plays a role in the Wnt signaling pathway, crucial for proper development [65,66].

The expression of HHEX was first studied in hematopoietic cells of birds and humans. It was also detected in cDNA libraries of fertilized human oocytes during the 2–8 cell division cycles. After gastrulation, Prh is expressed within the embryo in the mesoderm that gives rise to hematopoietic cells and vascular progenitor cells, parts of the endocardium that will develop into the heart, and in endodermal tissues involved in the formation of organs such as the liver, thyroid, lungs, thymus, gallbladder, and pancreas [64,66,67].

In mammals, the pancreas develops from the ventral and dorsal portions of the endoderm. After the organogenesis period, HHEX is expressed in the ventral portion of the embryonic pancreas. In Prh(-) embryos, there were disturbances in the morphogenesis of this portion of the pancreas, although gene regulation remained normal. HHEX regulates pancreatic development by controlling the proliferation of endodermal cells and their proper localization [68].

In adulthood, the activity of this gene has been observed in the lungs, thyroid, and liver, though studies have only been conducted in animal models. No expression of HHEX has been detected in the pancreas, nor has any function of the gene in this organ been demonstrated. J. Zhang’s team was the first to investigate the impact of the protein encoded by this gene on the endocrine function of the fetal and adult pancreas. They discovered that the expression of this protein occurs not in beta cells, as initially thought, but in delta cells that secrete somatostatin and regulate the function of alpha and beta cells in the pancreas. Delta cells constitute about 11% of all cells in the islets of Langerhans. In response to stimulation, such as by glucagon, they release somatostatin (Sst), which binds to receptor 2 (Sstr2) on alpha cells and receptor 5 on beta cells. This interaction results in the inhibition of glucagon and insulin secretion. Disruptions in HHEX expression, such as in T2D, may lead to abnormalities in the paracrine regulation of insulin secretion, resulting in accelerated depletion of beta cells [69].

Due to the role of HHEX in development and cellular regulation, research has focused on the relationship between genetic polymorphisms and the risk of developing T2D. The genetic variants studied are summarized in Table 2. Polymorphisms rs7923837, rs1111875, and rs5015480 are associated with the risk of T2D. The risk varies across different populations and is influenced by the selection of control groups.

For instance, the frequency of the rs1111875 allele is:

- 77.6% in the African population
- 61.97% in the Caucasian population
- 62.75% in the Mexican population
- 34.52% in the Asian population

Variants rs7923837, rs1111875, and rs5015480 are linked to the regulation of insulin secretion and beta cell function [70,71] (Table 2).

In an experiment conducted by A. H. Rosengren et al., it was demonstrated that the risk allele rs1111875 reduces the number of granules in beta cells, thereby impairing the ability to exocytose insulin [72]. These findings highlight the complex interplay between genetic variations in HHEX and their impact on diabetes risk and pancreatic function.

## 9. Discussion and Clinical Application

**Figure 3:**
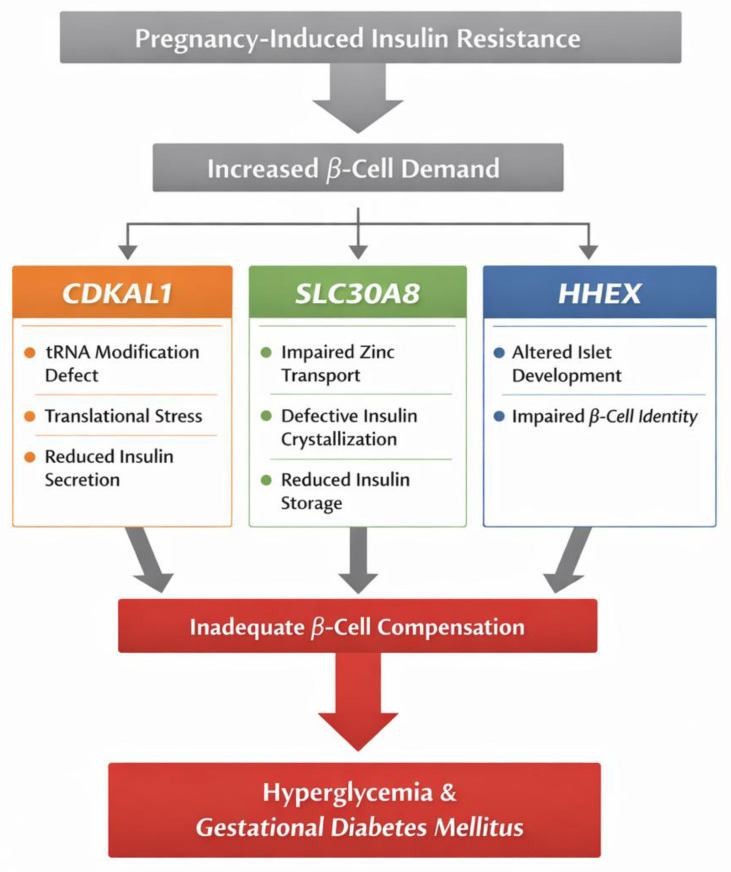
Integrated model of β-cell dysfunction in GDM involving CDKAL1, SLC30A8 and HHEX.

Our review synthesizes growing evidence that genetic variability in SLC30A8, CDKAL1, and HHEX contributes to susceptibility to GDM via mechanisms that primarily compromise β-cell function and the ability to meet the increased insulin demands of pregnancy. The cumulative genetic data consistently indicate that the dominant molecular axis underlying GDM is impaired insulin biosynthesis and secretion rather than isolated insulin resistance mechanisms. Although SLC30A8, CDKAL1 and HHEX act at different molecular levels, they converge on a single functional pathway—the capacity of pancreatic β-cells to synthesize, store and precisely regulate insulin secretion in response to metabolic demand. Together, they form a continuum from proinsulin translation (CDKAL1), through zinc-dependent granule maturation and storage (SLC30A8), to intra-islet paracrine control of secretion (HHEX), providing an integrated genetic framework for β-cell failure in GDM (Figure 3).

The strongest and most reproducible body of evidence concerns CDKAL1, as summarized across multiple independent cohorts and meta-analyses (Table S1). Genome-wide association studies conducted in Asian and European populations have demonstrated significant associations between CDKAL1 variants and GDM, particularly for intronic polymorphisms such as rs7754840, rs7756992, and rs10946398 [73,74]. These findings are supported by functional data showing that CDKAL1 is involved in post-transcriptional tRNA modification essential for accurate lysine incorporation into proinsulin. Impairment of CDKAL1 leads to defective insulin synthesis, reduced glucose-stimulated insulin secretion, and an attenuated first-phase insulin response [55,60,61]. Animal models lacking CDKAL1 exhibit abnormal mitochondrial function and reduced ATP generation in β-cells, reinforcing the importance of this gene in insulin biosynthesis [62,63]. However, population-specific differences remain evident, as not all ethnic cohorts demonstrate statistically significant associations, highlighting the complex genetic architecture of GDM [75].

SLC30A8 represents another major functional regulator with strong biological plausibility. The missense polymorphism rs13266634, encoding the R325W substitution in ZnT8, has been consistently associated with susceptibility to both T2D and GDM [72,73]. Meta-analyses indicate that the C (Arg) allele confers an increased risk of GDM, with the strongest and most consistent effects observed in Asian populations [73] (Table S1). ZnT8 plays a crucial role in zinc accumulation within insulin granules, which is required for insulin crystallization and storage. Experimental models demonstrate that impaired ZnT8 function leads to reduced insulin content, delayed insulin secretion, and dysregulated β-cell metabolism [41,42,43]. Importantly, studies indicate that dietary zinc intake may modulate the effect of SLC30A8 risk alleles on glycaemia, providing a potential nutrigenetic dimension to disease susceptibility.

Polymorphisms near HHEX exhibit a weaker but still consistent association with GDM (Table S1). Multiple meta-analyses report associations between rs1111875, rs5015480, and rs7923837 and GDM risk, particularly in East Asian cohorts [70,73] (Table S1). HHEX is essential for pancreatic development and maintenance of δ-cell function in the adult pancreas, with implications for paracrine regulation of insulin secretion through somatostatin signalling [69]. Functional studies demonstrate that risk alleles near HHEX reduce the number of insulin granules and impair exocytosis capacity in β-cells [72]. Despite clear physiological relevance, the mechanisms linking HHEX polymorphisms to GDM are less direct than for CDKAL1 or SLC30A8, suggesting that HHEX acts as a modulatory rather than primary determinant.

Importantly, GDM is a polygenic disorder and the effect size of individual loci remains modest. Recent reviews emphasize that genetic variants affecting placental insulin signalling, inflammation, and adipokine production further modify maternal metabolic adaptation [76,77]. Moreover, epigenetic regulation and environmental modifiers such as maternal obesity, micronutrient status, and gestational weight gain significantly influence genetic penetrance [78].

Among the three analyzed genes, CDKAL1 shows the most consistently replicated associations with GDM risk across independent cohorts, including large-scale GWAS and genetic association studies, as summarized in Table S1 [55,60,61,73,74]. Polymorphisms in CDKAL1 primarily impair proinsulin translation and first-phase insulin secretion, a central defect in GDM pathogenesis. SLC30A8 represents the second-most clinically relevant gene, particularly due to its potential interaction with maternal zinc availability and nutrition [41,42]. This raises the possibility of targeted dietary or supplementation strategies in genetically susceptible individuals. HHEX polymorphisms appear to exert a weaker but biologically meaningful effect, acting through altered β-cell regulation and paracrine control of insulin release rather than direct insulin biosynthesis [69,70,72].

A future clinical strategy could involve a polygenic risk panel including CDKAL1 and SLC30A8 variants to identify women at risk early in pregnancy. For high-risk genotypes, earlier glucose tolerance testing, intensified monitoring, and targeted nutritional interventions may serve as preventive strategies.

## 10. Conclusions

The reviewed evidence indicates that genetic susceptibility in gestational diabetes mellitus is primarily driven by defects in pancreatic β-cell capacity rather than by isolated disturbances in insulin sensitivity. Variants in SLC30A8, CDKAL1, and HHEX do not represent independent or random risk markers; instead, they converge on a single biological axis—the ability of the β-cell to meet the increased insulin demand of pregnancy. These genes act at successive and complementary stages of the insulin secretory pathway. CDKAL1 determines the fidelity and efficiency of proinsulin translation, thereby limiting the amount of functional insulin that can be produced. SLC30A8 governs zinc-dependent insulin granule maturation and storage, directly shaping the size and quality of releasable insulin pools. HHEX modulates intra-islet signaling, influencing paracrine control of insulin secretion through δ-cell-mediated regulation. These loci define a coherent molecular framework in which GDM emerges as a failure of β-cell compensation in the face of pregnancy-induced insulin resistance. Among them, CDKAL1 shows the most robust and consistently replicated association with GDM across populations, supported by strong functional evidence linking its variants to impaired first-phase insulin secretion. SLC30A8 represents a second key determinant, connecting genetic risk to a concrete cellular mechanism and potentially to modifiable environmental factors such as maternal micronutrient status. The effects of HHEX are weaker and more population-dependent, consistent with a modulatory rather than primary role.

The heterogeneity of genetic effects across ethnic groups highlights the importance of ancestry-specific analyses and standardized diagnostic criteria. Future research should integrate large multiethnic prospective cohorts with mechanistic studies to identify causal variants and quantify their functional impact. Only such evidence will determine whether genetic profiling can be translated into clinical practice to enable targeted screening or preventive strategies in women at highest biological risk of GDM.

## Figures and Tables

**Table 1:** Diagnostic criteria for GDM (a single glucose value above the threshold is sufficient for diagnosis).

	PTD (2011)	IADPSG/WHO 2013
Fasting	≥100 mg/dL (5.6 mmol/L)	≥92 mg/dL (5.11 mmol/L)
1 h of OGTT	≥180 mg/dL (9.99 mmol/L)	≥180 mg/dL (9.99 mmol/L)
2 h of OGTT	≥140 mg/dL (7.8 mmol/L)	≥153 mg/dL (8.49 mmol/L)

PTD—Polish Diabetes Association, IADPSG—International Association of Diabetes and Pregnancy Study Groups, WHO—World Health Organization, OGTT—Oral Glucose Tolerance Test, commonly used for diabetes diagnosis, Fasting—Measurement of blood glucose after fasting, 1 h of OGTT—Measurement of blood glucose 1 h after glucose administration in the tolerance test, 2 h of OGTT—Measurement of blood glucose 2 h after glucose administration in the tolerance test.

**Table 2:** Characteristics of genetic polymorphisms near HHEX.

NP	Chromosomal Location	Genotypic Frequency (HapMap-CEU) European Population	Allelic Frequency (HapMap-CEU) European Population	Primary Allele
rs7923837	92722160	AA: 0.159, AG: 0.416, GG: 0.425	A: 0.367, G: 0.633	G
rs5015480	92705802	CC: 0.322, CT: 0.492, TT: 0.186	C: 0.568, T: 0.432	C
rs1111875	92703125	AA: 0.168, AG: 0.496, GG: 0.336	A: 0.416, G: 0.584	A

For rs7923837, allele G is the primary allele and is more prevalent in the European population. For rs5015480, allele C is the primary allele and is more common in the European population. For rs1111875, allele A is the primary allele and has a higher frequency in the European population.

## Data Availability

No new data were created or analyzed in this study. Data sharing is not applicable to this article.

## Supplementary Materials

The following supporting information can be downloaded at: https://www.mdpi.com/article/10.3390/ijms27021121/s1. References [73,75,79,80,81,82,83,84,85,86,87,88,89,90] are cited in the Supplementary Materials.
