# Supplementary material for: Molecular Genetics of β-Cell Compensation in Gestational Diabetes Mellitus: Insights from CDKAL1, SLC30A8 and HHEX
Source: Int J Mol Sci. 2026 Jan 22;27(2):1121. doi: 10.3390/ijms27021121 (PMC12842414; doi:10.3390/ijms27021121)
Supplement: Supplementary file 1 [file ijms-27-01121-s001.zip › ijms-4087142-supplementary.pdf]

Table. S1 Summary of key genetic association studies evaluating SLC30A8, CDKAL1, and HHEX polymorphisms in gestational diabetes mellitus.

Reference (quality)	Population	GDM criteria	Cases/Controls	Gene	Variant	Effect (OR [95% CI], model)	Key covariates
Amin et al., 2022 (case–control; multiple models)	Asian	IASDPG	212 / 256	CDKAL1	rs7754840	Recessive model (CC vs CG+GG): OR=2.09; 95% CI 1.00–4.36. After adjustment OR=2.23; 95% CI 1.04–4.75	Adjusted for gravidity and family history of diabetes
Amin et al., 2022 (case–control; multiple models)	Asian	IASDPG	212 / 256	CDKAL1	rs7756992	Dominant model (AG+GG vs AA): OR=1.59 (95% CI 1.10–2.30), P=0.014; Log-additive: OR=1.42 (1.06–1.90), P=0.019.	Adjusted for gravidity and family history of diabetes
Cho et al., 2009 (case–control)	Asian	Carpenter–Coustan criteria	869/632	CDKAL1	rs7754840	1.55 [1.34–1.79], additive	Not reported / genetic association; cohort comparison
Cho et al., 2009 (case–control)	Asian	Carpenter–Coustan criteria	869/632	CDKAL1	rs7756992	1.39 [1.20–1.61], additive	Not reported / genetic association; cohort comparison
Cho et al., 2009 (case–control)	Asian	Carpenter–Coustan criteria	869/632	HHEX	rs1111875	1.27 [1.09–1.49], additive	Not reported / genetic association; cohort comparison
Cho et al., 2009 (case–control)	Asian	Carpenter–Coustan criteria	869/632	SLC30A8	rs13266634	1.24 [1.07–1.43], additive	Not reported / genetic association; cohort comparison
Gyan et al., 2025 (BMC Endocr Disord) – case-control, meta- analysis	Asian	IADPSG	500/502	CDKAL1	rs7754840 (G>C)	Dominant model OR 1.16 (1.104–1.29); Adjusted codominant model OR 1.715 (1.133–2.595)	Adjusted for age, pre-pregnancy BMI, SBP/DBP, parity
Lauenborg et al.,2009 (Danish prior-GDM cohort)	Caucasian	WHO	283/2446	CDKAL1	rs7756992	Additive model 1.22 [1.00–1.49]	Adjusted: age, BMI
Lauenborg et al.,2009 (Danish prior-GDM cohort)	Caucasian	WHO	283/2446	HHEX	rs1111875	Additive model 1.18 [0.98–1.43]	Adjusted: age, BMI
Lauenborg et al.,2009 (Danish prior-GDM cohort)	Caucasian	WHO	283/2446	SLC30A8	rs13266634	Additive model 1.19 [0.97–1.44],	Adjusted: age, BMI
Noury et al., 2018 (Egyptian cohort)	Middle Eastern/North African	IASDPG	47/51	CDKAL1	rs7754840	Allelic model 1.199 [0.669–2.152],	Not reported / unadjusted
Pervjakova et al., 2022 (multi-ancestry GWAS meta-analysis)	Multi-ancestry	multi-cohort	5485/347856	CDKAL1	rs9348441	Fixed-effects allelic model 1.13 [1.08–1.18]	GWAS standard covariates (study-specific; ancestry PCs etc.)
Pineda-Cortel et al., 2021; (case-control)	Asian	IASDPG	101 / 99	CDKAL1	rs7754840	1.43 [0.82–2.50]	NA
Rosta et al., 2017 ; (case-control)	Caucasian	IASDPG, WHO	IADPSG: 287/533; WHO: 303/517	SLC30A8	rs13266634	Allele T (OR=0.74 (IADPSG) and OR=0.71 (m'99 WHO); p=0.05/0.02 (C/T). CI not reported.	Adjusted for age and BMI (additive model)
Rosta et al., 2017 ; (case-control)	Caucasian	IASDPG, WHO	IADPSG: 287/533; WHO: 303/517	CDKAL1	rs7754840	Allele C : OR=1.51 (IADPSG) and NS under m'99 WHO; p=0.016. CI not reported.	Age-adjusted
Wang et al., 2011; (case-control)	Asian	Carpenter–Coustan criteria	725/1039	CDKAL1	rs7754840	Additive model OR 1.274 (0.957–1.695);	Not reported
Yu et al., 2021 (meta-analysis)	Asian	various	4,819 / 5,873	CDKAL1	rs7754840	CG vs GG: OR 1.36 [1.13–1.65]; CC vs GG: OR 1.76 [1.37–2.26]	Pooled unadjusted genetic models
Yu et al., 2021 (meta-analysis)	Asian	various	2,376 / 4,458	CDKAL1	rs7756992	AG vs AA: OR 1.23 [1.08–1.41]; GG vs AA: OR 1.47 [1.05–2.05]	Pooled unadjusted genetic models
Zeng et al., 2023; (age-stratified analysis)	Asian	IASDPG	500/502	SLC30A8	rs13266634	Recessive moedl 0.615 (0.392–0.966)	Adjusted for age, pre-pregnancy BMI, SBP/DBP, parity

Zeng et al., 2023; (age-stratified analysis)	Asian	IASDPG	500/502	SLC30A8	rs2466293	Adjusted dominant model OR 1.310 (1.005–1.707)	Adjusted for age, pre-pregnancy BMI, SBP/DBP, parity
Zeng et al., 2023; (age-stratified analysis)	Asian	IASDPG	500/502	HHEX	rs5015480	Adjusted dominant model 1.595 (1.034–2.459)	Adjusted for age, pre-pregnancy BMI, SBP/DBP, parity
Zeng et al., 2023; (age-stratified analysis)	Asian	IASDPG	500/502	HHEX	rs1111875	Adjusted heterozygote model 1.370 (1.040–1.805)	Adjusted for age, pre-pregnancy BMI, SBP/DBP, parity
Kwak et al., 2012 (GWAS)	Asian	Carpenter–Coustan criteria	468/1242	CDKAL1	rs7754840	1.707[1-459-1997], (p=<0.0001)	GWAS standard covariates
Xie et al., 2023 (meta-analysis).	Multi-ancestry	Various across included studies (IWCGDM, WHO, ADA, Carpenter–Coustan criteria, etc.)	1,232 / 4,284	HHEX	rs1111875	Allelic/additive (C vs T): OR 1.20 (1.07–1.34); P=0.001; I²=0%; fixed-effect (3 studies; 4 groups)	Pooled unadjusted genetic models (NOS ≥6 for included studies)
Xie et al., 2023 (meta-analysis).	Multi-ancestry	Various across included studies (IWCGDM, IADPSG, ADA)	1,384 / 1,184	HHEX	rs5015480	Allelic/additive (C vs T): OR 1.29 (1.13–1.47); P<0.001; I²=0%; fixed-effect (3 studies)	Pooled unadjusted genetic models (NOS ≥6 for included studies)
Xie et al., 2023 (meta-analysis).	Multi-ancestry	Various across included studies (IWCGDM, WHO, ADA, Carpenter–Coustan, EASD, SBD)	2,610 / 5,910	SLC30A8	rs13266634	Allelic/additive (C vs T): OR 1.23 (1.13–1.33); P<0.001; I²=13.6%; fixed-effect (7 studies; 8 groups)	Pooled unadjusted genetic models (NOS ≥6 for included studies)
Stuebe et al., 2014; prospective cohort	Caucasian/ African-American	Carpenter–Coustan criteria	Caucasian: 56/843; African-American: 24/362	CDKAL1	rs10946398	Caucasian: 1 risk allele vs 0 OR 0.75 [0.41–1.37]; 2 vs 0 OR 0.35 [0.04–1.44]. African-American: 1 vs 0 OR 0.83 [0.23–3.81]; 2 vs 0 OR 0.81 [0.20–3.97].	Adjusted for maternal age and pregravid BMI; among African-American women additionally adjusted for probability of Yoruban ancestry.
Stuebe et al., 2014; prospective cohort	Caucasian/ African-American	Carpenter–Coustan criteria	Caucasian: 56/843; African-American: 24/362	HHEX/IDE	rs1111875	Caucasian: 1 vs 0 OR 1.05 [0.71–1.55]; 2 vs 0 OR 1.10 [0.50–2.40]. African-American: 1 vs 0 OR 1.35 [0.16–64.99]; 2 vs 0 OR 1.45 [0.19–66.21].	Adjusted for maternal age and pregravid BMI; among African-American women additionally adjusted for probability of Yoruban ancestry.
Stuebe et al., 2014; prospective cohort	Caucasian/ African-American	Carpenter–Coustan criteria	Caucasian: 56/843; African-American: 24/362	SLC30A8	rs11558471	Caucasian: 1 vs 0 OR 1.79 [0.52–9.61]; 2 vs 0 OR 1.68 [0.49–9.02]. African-American: 1 vs 0 OR 0.15 [0.02–>999.99]; 2 vs 0 OR 0.29 [0.04–>999.99].	Adjusted for maternal age and pregravid BMI; among African-American women additionally adjusted for probability of Yoruban ancestry.
Stuebe et al., 2014; prospective cohort	Caucasian/ African-American	Carpenter–Coustan criteria	Caucasian: 56/843; African-American: 24/362	SLC30A8	rs13266634	Caucasian: 1 vs 0 OR 1.46 [0.48–5.99]; 2 vs 0 OR 1.48 [0.49–6.08]. African-American: 1 vs 0 OR 0.14 [0.02–>999.99]; 2 vs 0 OR 0.30 [0.04–>999.99].	Adjusted for maternal age and pregravid BMI; among African-American women additionally adjusted for probability of Yoruban ancestry.

Abbreviation: SBP/DBP -Systolic Blood Pressure / Diastolic Blood Pressure, IASDPG-International Association of Diabetes in Pregnancy Study Groups, IWCGDM -The International Workshop-Conference (IWC) on GDM, ADA- American Diabetes Association,

References :

1. Amin, U.S.M.; Parvez, N.; Rahman, T.A.; Hasan, M.R.; Das, K.C.; Jahan, S.; Hasanat, M.A.; Seraj, Z.I.; Salimullah, M. CDKAL1 gene rs7756992 A/G and rs7754840 G/C polymorphisms are associated with gestational diabetes mellitus in a sample of Bangladeshi population: implication for future T2DM prophylaxis. *Diabetol Metab Syndr*. **2022** ,*14*, 18.
2. Cho, Y.M.; Kim, T.H.; Lim, S.; Choi, S.H.; Shin, H.D.; Lee, H.K.; Park, K.S.; Jang, H.C. Type 2 diabetes-associated genetic variants discovered in the recent genome-wide association studies are related to gestational diabetes mellitus in the Korean population. *Diabetologia* **2009**, 52, 253–261.

3. Gyan, W. R.; Zhang, H.; Shao, T.; Yang, T.; Wei, Y.; Li, M.; Che, X.; Zeng, Q.; Guo, R. Association of CDKAL1 gene polymorphisms variations with gestational diabetes mellitus risk in women: A case-control study and meta-analysis. *BMC Endocr Disord.* **2025**, 25, 125.
4. Lauenborg, J.; Grarup, N.; Damm, P.; Borch-Johnsen, K.; Jørgensen, T.; Pedersen, O.; Hansen, T. Common type 2 diabetes risk gene variants associate with gestational diabetes. *J Clin Endocrinol Metab.* **2009**, *94*, 145-150.
5. Noury, A.E.; Azmy, O.; Alsharnoubi, J.; Salama, S.; Okasha, A.; Gouda, W. (2 Variants of CDKAL1 rs7754840 (G/C) and CDKN2A/2B rs10811661 (C/T) with gestational diabetes: insignificant association. *BMC Res Notes.* **2018**, 11, 181.
6. Pervjakova, N.; Moen, G.H.; Borges, M.C.; Ferreira, T.; Cook, J.P.; Allard, C.; Beaumont, R.N.; Canouil, M.; Hatem, G.; Heiskala, A., et al. Multi-ancestry genome-wide association study of gestational diabetes mellitus highlights genetic links with type 2 diabetes. *Hum Mol Genet.* **2022**, *31*, 3377-3391.
7. Pineda-Cortel; Maria, Ruth B.; Baybayan, Karlo; Bello, Peter Louie; Camenforte, Latiffa Lois; Ching, Stefany Jane; Conti, Kathleen; Ignacio, Jeremiah Jose; Diaz, Jiovanni; Villavieja, Adrian; et al. Genetic association of rs7754840 and rs7756992 polymorphisms in the CDKAL1 gene and gestational diabetes mellitus in selected Filipino pregnant women. *Asian Pacific Journal of Reproduction* **2021**, *10*, 155-161.
8. Rosta, K.; Al-Aissa, Z.; Hadarits, O.; Harreiter, J.; Nádasdi, Á.; Kelemen, F.; Bancher-Todesca, D.; Komlósi, Z.; Németh, L.; Rigó, J.; et al. Association Study with 77 SNPs Confirms the Robust Role for the rs10830963/G of MTNR1B Variant and Identifies Two Novel Associations in Gestational Diabetes Mellitus Development. *PLoS ONE* **2017**, *12*, e0169781.
9. Wang, Y.; Nie, M.; Li, W.; Ping, F.; Hu, Y.; Ma, L.; Gao, J.; Liu, J. Association of Six Single Nucleotide Polymorphisms with Gestational Diabetes Mellitus in a Chinese Population. *PLoS ONE* **2011**, *6*, e26953.
10. Yu, X.Y.; Song, L.P.; Wei, S.D.; Wen, X.L.; Liu, D.B. CDK5 Regulatory Subunit-Associated Protein 1-Like 1 Gene Polymorphisms and Gestational Diabetes Mellitus Risk: A Trial Sequential Meta-Analysis of 13,306 Subjects. *Front Endocrinol (Lausanne).* **2021**, *12*, 722674.
11. Zeng, Q.; Liu, J.; Liu, X.; Liu, N.; Wu, W.; Watson, R. G.; Zou, D.; Wei, Y.; Guo, R. Association between genetic polymorphisms and gestational diabetes mellitus susceptibility in a Chinese population. *Front Endocrinol (Lausanne).* **2024**, *15*, 1397423.
12. Kwak, S.H.; Kim, S.H.; Cho, Y.M.; Go, M.J.; Cho, Y.S.; Choi, S.H.; Moon, M.K.; Jung, H.S.; Shin, H.D.; Kang, H.M.; et al. A genome-wide association study of gestational diabetes mellitus in Korean women. *Diabetes.* **2012**, *61*, 531-541.
13. Xie, W.; Zhang, L.; Wang, J.; Wang, Y. Association of HHEX and SLC30A8 Gene Polymorphisms with Gestational Diabetes Mellitus Susceptibility: A Meta-analysis. *Biochem Genet* **2023**, *61*, 2203-2221.
14. Stuebe, A.M.; Horton, B.J., Chetwynd, E.; Watkins, S.; Grewen, K.; Meltzer-Brody, S. Prevalence and risk factors for early, undesired weaning attributed to lactation dysfunction. *J Womens Health (Larchmt).* **2014**, *23*, 404-412.
